# Pathology and Practical Medicine

**Published:** 1876-04

**Authors:** 


### IV. Pathology and Practical Medicine.

1. *Intestinal Obstruction due to a large Biliary Calculus.* Martin and Brouardel. (*Le Progrès Méd.*, No. 2.)

A woman, 78 years old, had obstinate constipation and vomiting of bile which soon become stercoraceous. There was no fever. Croton oil was administered with enemata, without effect. The pulse soon became small, 110 to the minute, eyes hollow, face choleriform, extremities cold, prostration profound. She could not rise in bed; hiccough supervened; the stomach tolerated ice only; the abdomen became distended, and through it the intestinal loops could be distinguished. Palpation was not very painful except at the level of the right iliac fossa and transverse colon, where sensibility was greatly exaggerated. The rectum was completely empty. Four large enemata were again tried and no evacuation followed.

The next day a voluminous cylindrical swelling was detected in the right inguinal fossa, which led to the supposition that the obstruction existed in the cœcum. Massage and more injections; an intense hæmorrhoidal congestion only followed.

The next morning the patient passed an indurated and voluminous cholesteric calculus. Ten other smaller concretions were passed during the day, and about six litres of a substance varying in consistency from softness to extreme hardness (scybala.) A few days sufficed to complete the cure.

Eight days after its expulsion, the large calculus weighed eighteen grammes and measured in millimetres forty-three, twenty-three and sixteen; circumference, nine.

It was thought it had passed directly into the intestine by the adhesive process, without traversing the biliary passages and the small intestine. The latter course would almost assuredly have produced a fatal result.

2. *Intermittent Broncho-Pneumonia.* Bourgade. (*Jour. de Méd. et de Chir.*, Feb., 1876.)

The disease has been observed when the hygienic conditions of countries where fever prevails have been modified by cultivation, etc. The physical signs of the disorder are quite like those of ordinary broncho-pneumonia, though perhaps less pronounced. Its onset is very insidious and accompanied by violent matutinal fever, which almost completely disappears during the intervals of the access. The disease may continue obstinately. It is sometimes terminated by pernicious fever, yet if it is correctly diagnosticated, it yields readily to the sulphate of quinia.

3. *Cardiac Chorea.* Boyer. (*Le Progrès Médical*, No. 52.)

Chorea, exclusive of rheumatism and the exanthematous fevers, may induce fatal endo-carditis, not merely the choreic palpitations of Spetzmüller and Benedikt. In Boyer's two cases, there were valvular vegetations but no embolism, which disproves the English theory that capillary embolism produces chorea, since the latter evidently precedes the cardiac lesion.

One of the two cases reported, which at the outset assumed the paraplegic form, disclosed, *post-mortem*, a dense adherent cerebral pia-mater and the cortical substance as friable as in general paraplegia, a white substance also friable, a recent cervical spinal meningitis, and, in the lumbar portion of the cord, an alteration of the gray substance, and the right antero-lateral column. Others have noticed meningeal and cortical alterations in chorea: Blache cites a case of purulent meningitis; Rokitansky has seen softening of the medulla; Demme, sclerosis of the latter; Frichard, congestion; Lockart Clarke, alteration of the medullary cells.

Hayem recalls the lesions of the protuberance of the cerebral convolutions of the central parts of the hemispheres, etc., noted in grave cases of chorea; none, however, is constant, unless it be meningo-rachidian hyperæmia, which is rather a consequence of the terminal asphyxia.

Charcot remarks that symptomatic hemichorea, preceding or following hemiplegia, seems to be connected with lesions of the posterior portions of the optic thalami, of the tubercula quadrigemina, of the upper segment of the cerebral peduncle, and the foot of the fan-shaped termination of the crura cerebri (*couronne rayonnante.*)

Tückwel has reported two cases of chorea with obliteration of the posterior optic arteries.

4. *Differential Diagnosis of Hysteria and Epilepsy.* Delasianne. (*L'Union Méd.*, No. 15.)

It is true that hysteria presents itself in various forms, but in a certain number of cases there seems to be a combination of hysterical and epileptiform symptoms. In epilepsy the manifestations are cerebral—the movement is toward the brain, whether the point of departure is central or peripheral. In hysteria, the movement is toward the organs of motion—the manifestations are exterior—the point of departure is in the abdominal viscera. Of course in hybrid cases a precise diagnosis is impossible, as the symptoms of each neurosis are present.

In one case the reporter observed a woman, 20 years old, become suddenly pale, cease speaking, totter and fall; then make the loudest cries and weep for twenty minutes. The movements were purely epileptic, or at least epileptiform, while the cry was hysterical. In another case reported, there was a mingling of cataleptic and hysterical phenomena, with alternate predominance of the two disorders.

5. *Slate-colored Tænia.* Laboulbène. (*L'Union Méd.*, No. 14.)

The general hue of these parasites is white or yellowish-white. A vigorous and intelligent Frenchman, 60 years old, who had lived many years in the United States and taken the part of the Confederacy in our late war, had experienced various nervous and epileptiform disorders; sudden aphasia without loss of consciousness, difficulty of articulation, dyspepsia, rheumatoid pains, etc.

He finally discovered blackish dry granules in his bed. Under the microscope and moistened, these proved to be proglottides of tænia with a genital pore on each side, but with this peculiarity, that a blackish pigment in the form of minute granules, was distributed over the superficial layers of the tissue. The patient's wife assured the reporter that the negresses of North America often passed black worms (!)

The decoction of pomegranate rind brought about the evacuation of a large, strong living tænia, but gray and slate colored. Placed in a glass of warm water the worm moved slowly and exhibited salient and swollen genital pores of a whitish color, which, upon the discolored surface of the rest of the body, showed like pearls. The four disks upon the head were distinctly black. It measured six metres in length. Neither Davaine de Méricourt nor the reporter had ever seen or heard of a similar species of pigmented tænia.

6. *Rheumatism and Traumatism.* VERNEUIL (*Jour de Méd. et de Chir.*, Feb., 1876.)

Three groups are considered: 1. Where the wound and the constitutional disorder occur simultaneously and without appearing to have reciprocal effects. 2. Where the traumatic injury sensibly modifies the evolution of the general disease. 3. Where the course of the surgical lesion presents anomalies attributable to the constitutional condition.

The influence of the rheumatic diathesis upon the march of traumatic lesions seems clear. The serous effusion, œdema, plastic exudation, simple or hæmorrhagic congestion, pain of various kinds (precocious traumatic neuralgia), are of frequent occurrence in wounded rheumatic patients. On the other hand, there are abundant examples of traumatic injury which seem to reawaken articular diseases long since relieved.

Verneuil cites the case of a patient who, after contusion of the hip, had coxo-femoral arthritis and rheumatic disorder of several distant articulations. This woman had never suffered from rheumatism, but presented articular deformities of the toes. In other cases, rheumatism with various complications has appeared in rheumatic subjects a few days before a fracture. The phenomena occurring in the joints were not the sole evidences of the disease. Others, such as eruptions and congestions of different organs were likewise observed.

Gasselin has been struck with the influence exerted by the rheumatic diathesis upon traumatic arthritis. In these cases there is no tendency to resolution but to stiffness and anchylosis. Verneuil points to premature movement and prolonged immobility as obstacles to cure, and believes that *rheumatic arthritis occurring in the wounded has been mistaken for purulent infection*, and the favorable result correspondingly misinterpreted.

7. *A New Form of Pseudo-Paraplegia.* DR. T. GRAINGER STEWART. (*Lancet*, Nov., 1875.)

A Scotch laborer, æt. 35, complained of want of power in both legs and the right arm, from which he had suffered three years. At the beginning he had felt the sensation of a tight band drawn about his waist, which for awhile had prevented him from standing erect. Later, he had a sensation of uneasiness passing up the left leg and down the right, at which point it felt as though boiling water was being poured upon the limb.

Now, his sensation was good except slight numbness in all the digits of his right hand, except the thumb and fore-finger. Sight had slightly failed, especially in the right eye. Micturition was slow, and the bowels were constipated. On tickling the soles the legs became rigid from spasm of the muscles. While he could grasp firmly with the right hand, he could not perform the finer muscular movements, as of writing. He could flex the thigh, but on attempting to flex the knees, ankles or toes the antagonistic muscles would come into play, the parts becoming rigid. On trying to walk the legs were rigid, and the feet had to be swung forward in a circle. Sitting down, going up stairs, and getting into bed, were very difficult, owing to the rigidity in the legs. The vaso-motor, cerebral and mental and nutritive functions were normal. The muscles were everywhere well nourished. Electro-sensibility and electro-contractility were normal.

The peculiarity of this case was, "the almost perfect soundness of the sensory functions, and the undue excitability of the motor structures," whereby voluntary movement was prevented by corresponding contractions of antagonistic muscles, "general contraction being also induced by peripheral irritation." These features, so far as Dr. S. is aware, do not correspond to any description of any disorder heretofore published. This patient was practically paraplegic although not really so, the power of muscular contraction being nowhere impaired. It must be a pseudo-paraplegia. The essentials of locomotor ataxy were wanting. It had none of the features of hysteria ; moreover, on examination, the discs of the eyes were grayish, their outline ill-defined, and the vessels were diminished in calibre—affording proof of organic disease of the cord. He believed the lesion was in the antero-lateral columns of the spinal cord, and that it was of the nature of sclerosis. He predicted the ultimate occurrence of real paraplegia.

The application of ice-bags along the spine appeared to be followed by temporary improvement. When they were applied for some time the patient bent his legs readily, but when he attempted to walk little difference was noticed. Ergot and conium had both proved useless.

8. *Infectious Pneumonia.* A. W. BLYTH. (*Lancet*, Nov., 1875.)

The author reports an epidemic during 1875 of pneumonia, about Barnstaple, England, that appeared to be infectious. The disease in many cases had symptoms of pleuritis, so that they might properly be called pleuro-pneumonia. In a number of instances a large part of a family would be attacked in succession. In the five unions (towns?) of South Molton, Okehampton, Torrington, Bideford, and Delverton, during 1874 the deaths from pneumonia per 1,000 deaths were respectively, 84, 48, 24, 22, 14. During the *first half* of 1875 the proportion was respectively, 126, 98, 111, 71, 144.

B. thinks the fact that cattle have an infectious pleuro-pneumonia; that both men and sheep have a small-pox; that both men and horses have a scarlet fever (strangles); that they both have a typhoid fever, should make us, by the mere suggestion of the analogy, ready to believe in the possibility of an infectious pleuro-pneumonia.

9. *Death by Lightning.* BALLANTI PIETRO. (*Il Raccoglitore Medico; Allg. Med. Centralz.*, 1876, No. 9.)

On Aug. 5, three laborers who had sought protection from a storm under a high pile of wheat were killed by lightning. The three bodies were found lying on their backs in a half circle, with dark blood flowing from the mouth and nostrils. The hair of the head was singed along the sagittal and lambdoid sutures; the hair of the chin, the breast, the arms and legs was entirely burnt off. The skin of the front of the bodies showed a number of small burns running in zig-zag lines. *Very peculiar to all three bodies was a red brown spot and a horizontal rent of the sclerotic coat of the right eye, as though it had been cut into.* Subcutaneous extravasation over the skulls; venous congestion of the meninges, cerebrum, cerebellum, and medulla oblongata; a sanious exudation at the basis of the brain and over the spinal cord. The right lung was normal, the left filled with dark blood. The right auricle and the left ventricle of the heart were filled with dark blood, while the left auricle and right ventricle were empty.

10. *Otis' Simplified Aspirator.* DR. F. N. OTIS. (*The Med. Record,* Jan. 29, 1876.)

Dr. O. has devised an aspirator "from materials within the easy reach of every surgeon." It "consists of an ordinary well-fitted syringe of any convenient size, joined to which by an inch of rubber tubing is a short hard rubber, metal or glass bifurcating tube. To each arm of this tube is attached another bit of rubber tubing which terminates in a hard rubber valve, such as is used in the construction of Davidson's syringe. These valves are arranged to work in reverse directions. On retraction of the piston, the valve which should be connected with the aspirating needle opens and permits access through the needle to the barrel of the syringe. The piston being driven back, this valve closes while the other opens and permits the escape of the fluid through the opposite tube, connected with a discharge pipe. The last mentioned valve is closed on the retraction of the piston. Near the base of the needle a short glass tube is inserted in a break in the tubing, to afford early information of the character of the fluid in process of evacuation."

11. *Mortality of Mariners, Railroad Men, etc.* EDGAR HOLDEN, M.D., Ph.D. (*Amer. Jour. Med. Sc.*, Jan., 1876.)

Dr. H. makes a careful study of the rate of mortality both from accident and natural causes in mariners, railroad employes and travelers for pleasure and business. It appears that the healthfulness of the occupation of railroad men, who are much in the open air, largely counterbalances the tendency to mortality from accident.

From a careful study of many tables of mortality of insurance companies, of railroads, and of the census, he concludes: "that the actual death-rate among railroad employes is less than among many occupations considered more favorable to life; that the *proportion* of deaths from the accidents of their occupation is considerable, but shows a balance favorable to the general healthfulness of the occupation; that the actual extra hazard lies among freight brakemen, but is yet less than that found to exist in the safer occupations."

The mortality among ordinary mariners is much greater, 12 or even 20 extra deaths per 1,000 "will not cover the actual extra mortality." This does not apply, however, to officers or seamen of our navy, whose death-rate "*from all causes* does not exceed that of the cities of the United States."

Among sailors in the bunks of coasting and fishing vessels, there was a mortality of 111 per 1,000, while in our navy in the years before the war, the mortality was only 20 per 1,000, reckoning officers and men together. To be more exact, among officers the rate was 25, and among the men 19 per 1,000.

The statistics of deaths from railroad accidents are interesting. In 1868 the average number of passengers carried to each one killed, on the railroads of the State of New York, was 236,337. In Great Britain, according to the report of the Board of Trade, "only one passenger out of 2,312,533 is said to have been killed." "In 1867 the figures stand 1 to 1,336,728; and, in 1866, 1 to 1,073,804."

"Voyagers for health or pleasure," Dr. H. concludes, "are not of necessity at extra risk of life," while voyagers for business or profit are at extra risk "only so far as the character of the climate, the length of stay, and question of acclimation are concerned."

12. *Vomiting of a Curious Worm.* SAGER. (*Peninsula Med. Jour.*, Feb., 1876.)

At a recent meeting of the Washtenaw Co. Medical Society, Dr. Abram Sager, of Ann Arbor, presented a photograph of a worm with the following history: "This worm, 2½ inches long, was vomited, while alive, from the stomach of a child five years old after taking a dose of ol.

terebinth. The child had heretofore been subject to feverish attacks which had been attributed to worms, and had been relieved by the use of common vermifuges. At this time the child had been indisposed for several days and passed several of the common ascaris lumbricoides, but got no relief until this worm was thrown off. The worm has bony mandibles, but no antennæ discoverable."

Dr. Sager thought it a larva of the common moth, and that it might subsist and grow for a time in the human stomach. There was always air enough in the stomach for the respiration of such an animal; it could live and breathe similar to the larvæ of the gadfly or bots in the stomach of the horse; "it could breathe through one of its spiral air tubes, there being free anastomosis between its air vessels, while attached to the mucous membrane of the stomach."

13. *A Great Mass of Cherry Stones in the Rectum.* DR. W. H. WESCOTT. (*Boston Med. and Surg. Jour.*, Feb. 24, 1876.)

A boy eight years old had, in July, complained of tenesmus for several days, but had passed only mucus tinged with blood. Cathartic medicine had produced only griping and more tenesmus. The bladder was found enormously distended and on attempting to pass the catheter, it was suddenly arrested. The rectum was found full of cherry-stones, "filling the whole pelvic cavity and pressing the urethra against the pubic arch." The stones were so firmly glued together that it was difficult to separate them. After removing with the forceps one hundred of these bodies, the boy passed urine freely. "Palpation discovered numerous masses of the stones in the large intestines," and the next day the rectum was again packed full of them. All of the stones discharged were not preserved, but those that were, measured six ounces and six drachms.

14. *Ready Method of Preparing Sections for the Microscope.* DR. JOHN STEVENSON. (*The Edinburg Med. Jour.*, Jan., 1876.)

A mixture of glycerine and tragacanth soon becomes stiff like jelly, and may be used to advantage in which to imbed tissues for the purpose of making slices for the microscope. It cuts like cheese after standing eight or nine hours, and by keeping it in methylated spirit twelve to twenty-four hours it parts with its glycerine and becomes more easily sliced by reason of its being harder. The material is dissolved off the section by cold water with a little glycerine added.

The proportion Dr. S. uses, is two drachms of glycerine to one and one-half drachms of powdered gum tragacanth—to be rubbed together on a slab or plate. Much less gum trag. than this proportion makes a material too soft. If not to be sliced within twelve hours from the time of its preparation the material should be preserved in methylated spirit.

15. *Significance of the Anæsthesia of the Deltoid Region in Dislocations of the Shoulder.* TH. ANGER. (*France Médicale*, 1876, No. 9.)

At a meeting of the "Société de Chirurgie," Anger reported the following case: A man contracted a dislocation of the shoulder by a fall; the next morning the doctor discovered a complete anæsthesia of the skin of the deltoid region and therefrom inferred a contusion or a laceration of the circumflex nerve had occurred. After reduction the anæsthesia persisted and the deltoid muscle was paralyzed. Six days afterwards the patient died, and the autopsy revealed a contusion of the circumflex nerve at the point where it gives off the cutaneous branch for the deltoid region; at that place there was an ecchymosis in the sheath of the nerve. When, therefore, before or after the reduction of a dislocated shoulder the sensation of the deltoid region is gone, one may fairly assume a lesion of the circumflex nerve and expect sooner or later to find the deltoid muscle paralyzed.

